# Subcutaneous Lobular Capillary Hemangioma Presenting as a Facial Mass

**DOI:** 10.1155/2018/5973619

**Published:** 2018-05-08

**Authors:** Charles Saadeh, Seckin O. Ulualp, Dinesh Rakheja

**Affiliations:** ^1^Department of Otolaryngology, Head and Neck Surgery, University of Texas Southwestern Medical Center, Dallas, TX, USA; ^2^Division of Pediatric Otolaryngology, Children's Health, Dallas, TX, USA; ^3^Division of Pathology, Children's Health, Dallas, TX, USA

## Abstract

Lobular capillary hemangioma is a benign lesion of the skin and mucous membranes. Subcutaneous lobular capillary hemangioma presents as a deeper nodule. Lack of the characteristic surface changes of this subtype of lobular capillary hemangioma makes the clinical diagnosis challenging. We describe clinical, radiologic, and histological features of a subcutaneous lobular capillary hemangioma tissue presenting as a facial mass in a 12-year-old male. The mass was a firm, nontender, immobile, subcutaneous nodule, with no color change of the overlying skin. CT imaging documented a hyperdense and nonlipomatous mass involving soft tissue of the left lateral nasal wall. An excisional biopsy was performed. Histologic evaluation showed subcutaneous lobular capillary hemangioma. Subcutaneous lobular capillary hemangioma, although uncommon, should be considered in the differential diagnosis of lateral nasal wall mass in children.

## 1. Introduction

Lobular capillary hemangioma (LCH), characterized by benign proliferation of capillaries with a lobular architecture, is a common benign vascular tumor of children and adults [1]. The International Society for the Study of Vascular Anomalies includes LCH in the benign vascular tumor group [1]. LCH may occur at all ages; however, LCH is more frequently seen in women and men under 18 years of age [2]. The most common etiologic factors are trauma, hormonal factors, and poor oral hygiene.

The majority of LCH present on the head and neck, commonly involving gingiva, lips, tongue, and buccal mucosa. LCH is rarely located in the nasal cavity [3]. The anterior septal mucosa, tip of the turbinate, and the vestibule are the common nasal cavity sites for LCH [4]. LCH may involve superficial cutaneous, mucosal, and subcutaneous structures. The rare occurrence and deep location of subcutaneous variant makes the clinical diagnosis challenging. We conducted a retrospective chart review to report the clinical, radiologic, and histological features of LCH in a child with a subcutaneous lateral nasal mass.

## 2. Case Report

**Figure 1:**
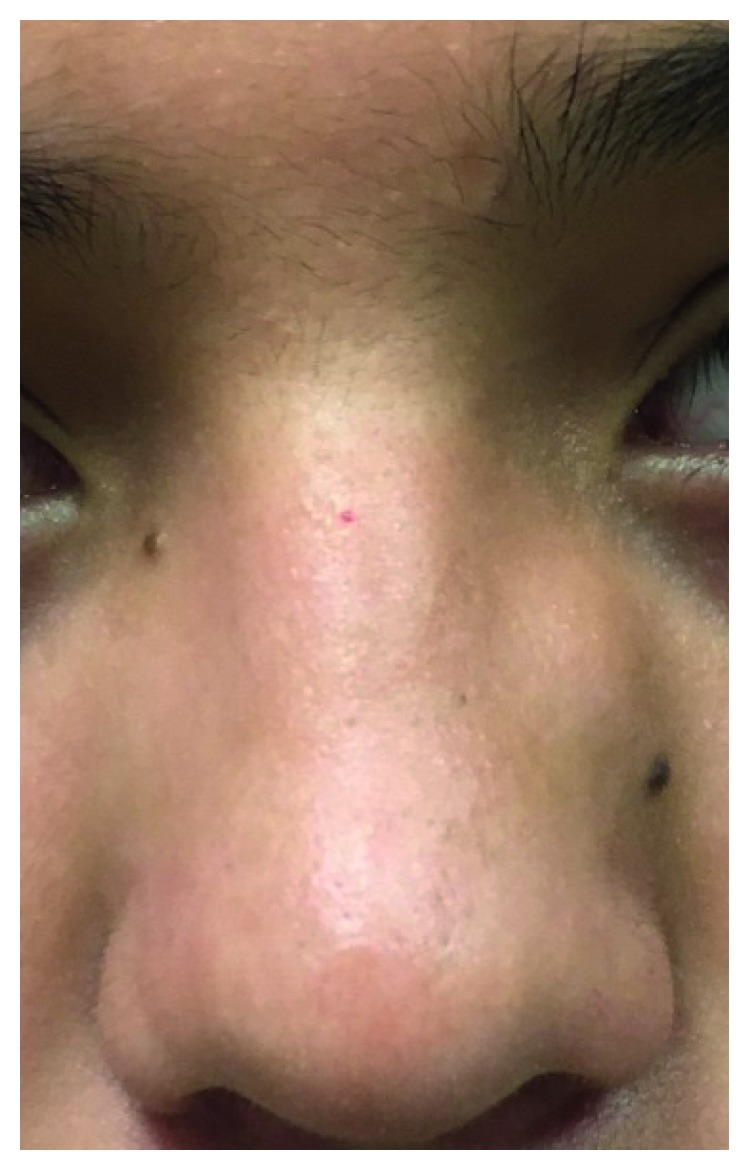
Subcutaneous mass with no color change of the overlying skin in the region of the frontal process of the maxilla.

**Figure 2:**
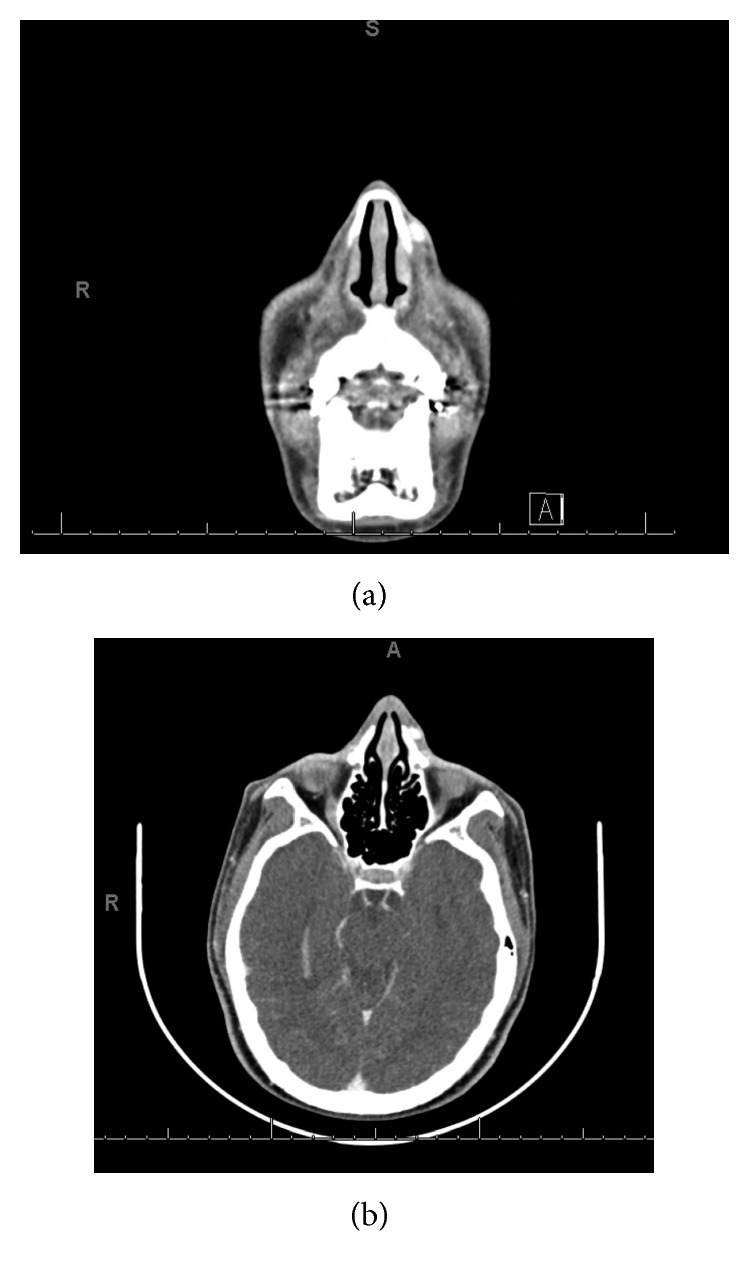
Axial contrast-enhanced CT images showing a well-demarcated, hyperdense, and nonlipomatous mass involving soft tissue of the left lateral nasal wall.

**Figure 3:**
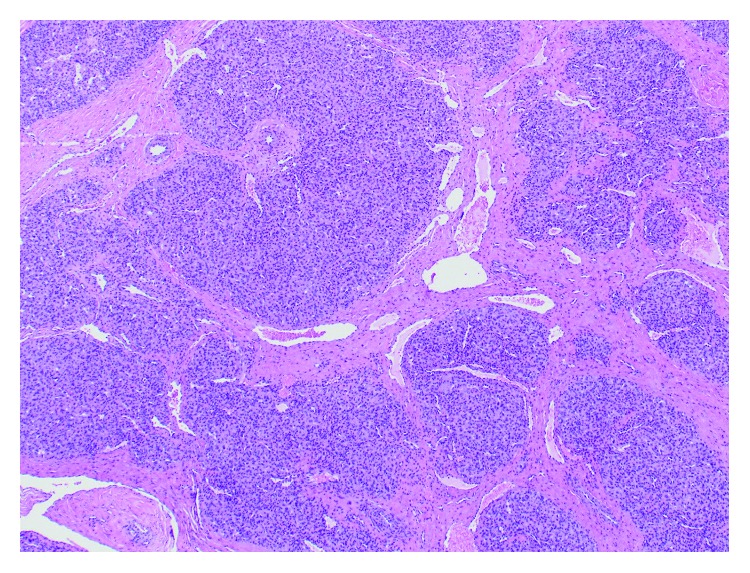
Photomicrograph of the excised specimen showing a proliferation of capillaries arranged in lobules separated by fibrous septa (hematoxylin and eosin, 40× original magnification).

A 12-year-old male with past medical history of type II diabetes mellitus presented with a gradually enlarging mass on the left lateral nasal wall for 8 months (Figure 1). The patient hit his nose on his brother's head a year ago. The child had no nasal obstruction, no epistaxis, no recurrent sinus infections, no anosmia nor hyposmia, and no snoring. There was no family history of childhood cancers. On physical examination, the 1.5 × 1.5 cm mass was a firm, nontender, immobile, subcutaneous mass in the region of the frontal process of the maxilla. There was no color change of the overlying skin. The patient presented to clinic with a magnetic resonance imaging (MRI) that did not show the mass due to artifact. Limited MR imaging suggested possible nasal dermoid. Computerized tomography of the mass documented a hyperdense and nonlipomatous mass involving soft tissue of the left lateral nasal wall (Figure 2). An excisional biopsy was performed. There was no apparent intranasal extension. Upon resection, histologic evaluation showed subcutaneous lobular capillary hemangioma (Figure 3). At 3 month follow-up, the surgical site was healed with no evidence of recurrent lesion.

## 3. Discussion

In the present study, subcutaneous LCH was documented in a child with a history of trauma to the nose. While no direct causal relationship has been identified, there is an association with estrogen exposure and trauma. Five percent of pregnant patients experience a new onset LCH, especially of the gingiva, hence the term “pregnancy tumor” [5]. Exogenous hormone therapy has also been implicated as a risk factor. Additionally, receptors to the beta isoform of estrogen have been identified on various vascular tumors, including hemangiomas, possibly supporting a hormonal etiology [6]. Prior local trauma is also frequently reported in the patient history, as was in this case; however, it is not considered necessary for development [5].

Histologically, LCH consists of a benign proliferation of capillaries arranged in lobules separated by fibrous septa [7]. Superficial lesions may ulcerate and be associated with inflammation and edema, while the deeper lesions often lack inflammation. The historical terminology, pyogenic granuloma, is a misnomer as the mass is neither infectious or pus producing nor granulomatous. Therefore, the current terminology of LCH is recommended. While usually not needed, immunohistochemistry for endothelial markers CD31 and Factor VIIII may be helpful.

The variants of LCH include oral mucosal, satellite, intravenous, dermal, and subcutaneous. Subcutaneous, as described in this case, is the rarest form of LCH and lacks the distinct friable, raised, easily bleeding appearance that is characteristic of the more common counterpart. A review of 106 LCH cases at a single institution found an incidence of 3.4% for the subcutaneous variant [5]. The clinical differential diagnosis of subcutaneous LCH includes a wide variety of subcutaneous pathologies, including but not limited to vascular malformation, Kaposiform hemangioendothelioma, and infantile hemangioma. The present case must also take into account the differential diagnosis for a child with a nasal sidewall mass. The clinical differential diagnosis of lateral nasal wall mass includes nasal dermoid, encephalocele, neoplasm, vascular malformation, and other various reported entities such as foreign body inclusion cyst and heterotopic glial tissue [8, 9]. Surgical treatment with complete excision is the mainstay of treatment and also provides definitive diagnosis. Recurrence, occurring in 16%, is due to incomplete excision of the mass [4, 10]. No malignant change has been reported [4].

In conclusion, subcutaneous mass can manifest as a lateral nasal wall mass in children, and excision of the mass forms the mainstay of treatment. Prompt diagnosis and treatment of subcutaneous LCH is essential to prevent complication or sequela.
